# Comparison of Pathogenicity and Transmissibility of Influenza B and D Viruses in Pigs

**DOI:** 10.3390/v11100905

**Published:** 2019-09-27

**Authors:** Jinhwa Lee, Liping Wang, Rachel Palinski, Tim Walsh, Dongchang He, Yonghai Li, Rui Wu, Yuekun Lang, Sun-Young Sunwoo, Juergen A. Richt, Wenjun Ma

**Affiliations:** Department of Diagnostic Medicine/Pathobiology, College of Veterinary Medicine, Kansas State University, Manhattan, KS 66506, USA; jinhwa@vet.k-state.edu (J.L.); lipingw@vet.k-state.edu (L.W.); walsht@vet.k-state.edu (T.W.); virolog@outlook.com (D.H.); yli@vet.k-state.edu (Y.L.); wurui1977@sicau.edu.cn (R.W.); yuekun@ksu.edu (Y.L.); sunwoosy@gmail.com (S.-Y.S.); jricht@vet.k-state.edu (J.A.R.)

**Keywords:** influenza B virus, influenza D virus, pathogenicity and transmissibility, pigs

## Abstract

Influenza viruses are important pathogens causing respiratory disease in humans and animals. In contrast to influenza A virus (IAV) that can infect a wide range of animal species, other influenza viruses, including influenza B virus (IBV), influenza C virus (ICV), and influenza D virus (IDV) have a limited host range. Swine can be infected with all four different genera of influenza viruses. IAV infection of pigs causes the well-known swine influenza that poses significant threats to human and animal health. However, influenza virus infection of pigs with IBV, ICV, and IDV are not well-characterized. Herein, we compared pathogenicity of IBV and IDV using intratracheal and intranasal infection of pigs, which are IAV seropositive, and commingled naïve pigs with the infected animals to determine their transmissibility. Both viruses caused fever and some lung lesions, replicated in the lungs of infected pigs, but only IDV transmitted to the contact animals. Although IBV and IDV displayed differing levels of replication in the respiratory tract of infected pigs, no significant differences in pathogenicity of both viruses were observed. These results indicate that both IBV and IDV can replicate, and are pathogenic in pigs.

## 1. Introduction

Influenza viruses are classified into four genera, including influenza A virus (IAV), influenza B virus (IBV), influenza C virus (ICV), and influenza D virus (IDV), which belong to the *Orthomyxoviridae* family [1,2]. IAV and IBV consist of eight negative-sense RNA segments, whereas ICV and IDV have only seven RNA segments. IAV and IBV cause severe seasonal human epidemics worldwide, resulting in 3000 to 48,000 deaths in the US each year, while ICV is merely associated with mild and asymptomatic respiratory disease in humans, particularly in children [3,4,5]. Although IDV specific antibodies were detected in humans [6], no virus has been isolated so far. Unlike IAV which has genetically distinct subtypes based on 18 hemagglutinin (HA) and 11 neuraminidase (NA) surface glycoproteins, IBV is classified into two antigenically and genetically distinct lineages: the Victoria-like lineage (B/Victoria/2/1987) and the Yamagata-like lineage (B/Yamagata/16/1988) [7,8]. In contrast, six genetically discrete lineages of ICV have been identified [9], while IDV is phylogenetically classified into three clusters based on the hemagglutinin-esterase (*HE*) gene [10].

Pigs are highly susceptible to IAV, which together with other pathogens such as porcine reproductive and respiratory syndrome virus (PRRSV), porcine circovirus type 2 (PCV2), *Mycoplasma hyopneumoniae*, *Bordetella bronchiseptica*, and *Actinobacillus pleuropneumoniae* cause porcine respiratory disease complex (PRDC), resulting in significant economic losses annually for swine industry [11]. Importantly, swine are known as the mixing vessel for multiple IAVs to generate novel reassortant strains that have the potential to infect humans and cause pandemics [12,13,14]. IAV infections in pigs are widespread and still represent an enormous challenge for human and animal health due to their rapid and frequent genetic changes. All influenza genera are capable of infecting pigs, although the role of IBV, ICV, and IDV in PRDC or the reassortment potential of these strains in pigs has not been determined.

In contrast to IAV, IBV lacks antigenic diversity and has limited gene reassortment; therefore it has not been implicated in influenza pandemics [15]. Despite the lack of pandemic potential, IBV is highly prevalent in patients with flu-like symptoms and may be associated with central nervous system complications, myositis, and even fatality in infected individuals [16,17]. IBVs have also been isolated from other animals, such as dogs, pheasants, and seals [18,19,20,21]. Previous studies have shown that antibodies against IBV have been detected in domestic pigs, and pigs are susceptible to IBV infection under experimental conditions [22,23,24]. In addition, IBVs were isolated from nasal swabs of naturally infected pigs in 2014 [25]. Taken together, swine may serve as the natural host and reservoir of IBVs.

ICV commonly infects humans. ICV has been isolated from naturally infected pigs and has been shown to experimentally infect and transmit among pigs [26]. IDV is a newly emerging genus of influenza virus, which was isolated from pigs with respiratory illness in Oklahoma in 2011, and has been proposed as a new genus of the *Orthomyxoviridae* family due to its genetic dissimilarity to other influenza viruses [2,27]. Subsequent studies regarding epidemiology and pathogenesis revealed that bovines are the primary natural host of IDVs and that IDVs circulate worldwide [28,29,30,31,32,33,34,35]. Furthermore, surveillance studies identified antibodies against IDV in sheep, goats, equines, and camels [36,37,38,39]; and human serum samples were also positive for IDV-specific antibodies with particularly high seroprevalence in persons occupationally exposed to cattle [6,27].

Although IBV, ICV, and IDV are capable of infecting swine and have been isolated from naturally infected pigs, the pathogenicity and transmissibility of these viruses have not been well characterized. In this study, we compared the pathogenicity and transmissibility of IBV and IDV after experimental infection of pigs.

## 2. Materials and Methods

### 2.1. Ethics Statement

The animal study was reviewed and approved by the Institutional Animal Care and Use Committee at Kansas State University (IACUC#4020, approved on December 13, 2017) and was performed in Biosafety Level 2+ animal facilities under guidance from the Comparative Medicine Group at Kansas State University.

### 2.2. Cells and Viruses

Madin–Darby canine kidney (MDCK) and swine testicle (ST) cells were cultured in Dulbecco’s modified Eagle medium (DMEM) supplemented with 5% fetal bovine serum (FBS) and 1% antibiotic-antimycotic (Invitrogen, Waltham, Massachusetts, USA). DMEM infecting media containing 0.3% bovine serum albumins (BSA) (Sigma-Aldrich, St. Louis, Missouri, USA), 1% antibiotic-antimycotic, and 0.5 or 1 μg/mL of N-tosyl-L-phenylalanine chloromethyl ketone (TPCK)-treated trypsin (Sigma-Aldrich) were used for virus infection of cells. Influenza B/Brisbane/60/2008 (Victoria lineage) was provided by the Centers for Disease Control and Prevention (CDC). Influenza D/bovine/Kansas/1-35/2010 was isolated from bovine nasal swab samples [30]. IBV was amplified in MDCK cells using DMEM infecting media containing 1 μg/mL TPCK-treated trypsin at 33 °C with 5% CO_2_ for 4 days. IDV was propagated in ST cells using DMEM infecting media containing 0.5 μg/mL TPCK-treated trypsin at 37 °C with 5% CO_2_ for 4 days. Virus loads in nasal swabs and bronchoalveolar lavage fluid (BALF) samples were determined on MDCK cells by indirect immunofluorescence assay (IFA). A 50% tissue culture infective dose (TCID_50_) was calculated to determine virus titers using Reed and Muench method [40].

### 2.3. Pigs

Thirty-five 5-week-old pigs were used in the study. Prior to the pig study, blood and nasal swab samples collected from each pig were used for serology and real-time RT-PCR tests. The pigs were seronegative to porcine reproductive and respiratory syndrome virus (ELISA assay), influenza B, and D viruses (HI assay), but had antibodies to the 2009 pandemic H1N1 (HI titer from 40-160) and swine influenza cluster IV H3N2 (HI titer from 40-640) viruses. All pigs were negative for influenza A, B, C, and D viruses by testing nasal swab samples using genus-specific real-time RT-PCR assays.

### 2.4. Experimental Design

Thirty-five pigs were randomly distributed into 3 groups including two infected groups and one control group. Each infected group contained 10 pigs and each pig was infected intratracheally (1.5 mL) and intranasally (1 mL) with 2.5 × 10^6^ TCID_50_ of B/Brisbane/60/2008 or D/bovine/Kansas/1-35/2010 virus, while the negative control group had 9 pigs that were mock-inoculated with 2.5 mL of DMEM media using the same routes. At 2 days post-infection (dpi), three contact pigs were commingled with the infected pigs in each group to investigate virus transmission. Body temperatures and clinical signs were monitored daily. Nasal swabs were collected from infected pigs at 0, 2, 4, 6, and 8 dpi and from contact pigs at 0, 2, 4, and 6 days post-contact (dpc) to determine virus shedding. Three pigs from each infected group and control group were necropsied at 4 and 6 dpi, and the remaining 4 infected pigs in each infected group and 3 mock-inoculated pigs were necropsied at 8 dpi. All contact pigs were necropsied at 6 dpc. Blood samples were collected from each pig prior to infection and at the necropsy day. During necropsy, bronchoalveolar lavage fluid (BALF) samples were collected by flushing each lung with 50 mL of fresh DMEM. Virus titers in nasal swabs and BALF samples were determined on MDCK cells.

### 2.5. Evaluation of Postmortem Tissues 

At necropsy, lungs were removed *in toto* and a single experienced veterinarian evaluated the percentage of typical influenza virus infection associated gross lesions of each lobe (each lung lobe is considered as 100%) as described previously [41,42]. The average of gross lung lesions of seven lung lobes of each pig was determined. After collection of BALF samples, lung and nasal turbinate specimens from each pig were collected and fixed in 10% neutral buffered formalin and routinely processed for histopathologic examination at the Kansas State Veterinary Diagnostic Laboratory. Lung and turbinate sections were examined by a board-certified veterinary anatomic pathologist in a blinded fashion. Lung scoring was assessed based on criteria described in a previous publication [43].

### 2.6. Indirect Immunofluorescence Assay and Real-Time PCR Assay

To determine virus titers in nasal swabs and BALF, an indirect immunofluorescence assay (IFA) was performed. MDCK cells in 96-well plates that were infected with serially diluted samples were fixed with methanol for 10 min followed by drying. After moistening the fixed plates with PBS, the plates were incubated with 5% FBS in PBST (PBS + 0.05% Tween 20) for 1 h at room temperature to block non-specific binding of the antibodies. A volume of 50 μL of mouse monoclonal anti-IBV nucleoprotein antibody (diluted 1:1000 with 1% FBS in PBST; Abcam, Cambridge, Massachusetts, USA) or pig polyclonal primary antibody against bovine IDV (diluted 1:200 with 1% FBS in PBST) was added to each well and incubated for 1 h at 37 °C. Following three times washing with PBST, the plates were incubated with a volume of 50 μL FITC conjugated goat anti-mouse IgG secondary antibody (diluted 1:200 with 1% FBS in PBST; Invitrogen, Waltham, Massachusetts, USA) or FITC conjugated rabbit anti-pig IgG secondary antibody (diluted 1:600 with 1% FBS in PBST; Invitrogen) for 1 h at 37 °C. After decanting secondary antibodies, plates were washed three times with PBST. Fluorescence signals were observed under a fluorescence microscope.

To detect IBV or IDV RNA in nasal swab and BALF samples, the specific real-time RT-PCR assays were performed. For detecting the IBV RNA, the CDC developed real-time RT-PCR assay targeting flu B *NS* gene was used [44], and the cutoff threshold cycle (Ct) value lower or equal to 38 was judged as positive; whereas to detect the IDV RNA, a specific real-time RT-PCR assay described previously targeting the flu D *PB1* gene was employed [27], and its cutoff Ct value lower or equal to 37 was judged as positive. RNA from nasal swab and BALF samples was extracted using QIAamp Viral RNA Mini Kit (Qiagen, Germantown, Maryland, USA) and the real-time RT-PCR was performed using the qScript XLT 1-Step RT-qPCR ToughMix (Quantabio, Beverly, Massachusetts, USA) according to the manufacturer’s instructions.

### 2.7. Hemagglutination Inhibition Assay

To perform the hemagglutination inhibition (HI) assay, serum samples were collected from infected pigs at 8 dpi and contact pigs at 6 dpc. HI assays were performed using 0.5% turkey red blood cells (RBCs) according to the standard protocol. Sera were treated with receptor-destroying enzyme (Denka-Seiken, Stamford, Texas, USA) overnight at 37 °C and 50% of turkey RBCs for 1 h to destroy nonspecific hemagglutination inhibitors and agglutinins. Serial two-fold dilution was performed for receptor-destroying enzyme-treated sera from 1:10 initial dilution. Diluted sera were incubated with 4 hemagglutinating units of IBV (B/Brisbane/60/2008) or IDV (D/bovine/Kansas/1-35/2010) for 30 min followed by incubation for 40 min with 0.5% turkey RBC solution.

## 3. Results

### 3.1. Pathogenicity of IBV and IDV in Pigs 

**Figure 1:**
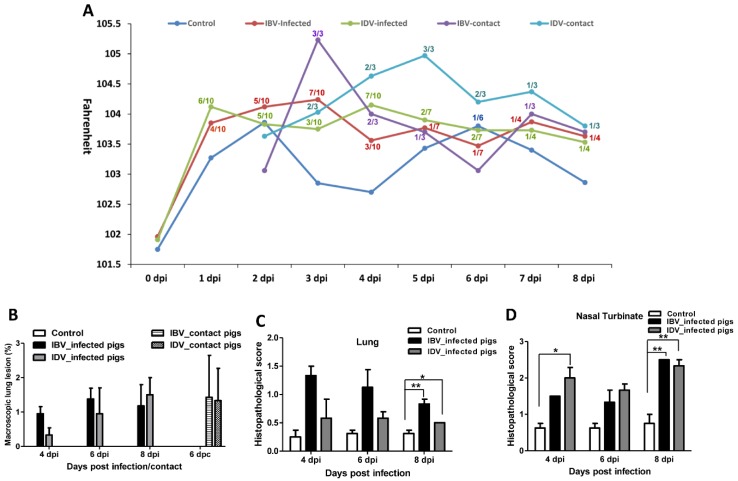
Body temperature, macroscopic, and microscopic lungs lesions of contact or infected pigs with influenza B virus (IBV), or influenza D virus (IDV). (**A**) The body temperature of infected or contact pigs is presented as the average body temperature of each group of pigs at indicated days. The number, such as 6/10, means that 6 out of 10 IDV-infected pigs showed fever at a defined dpi. (**B**) Macroscopic lung lesions of infected or contact pigs are presented as the average percentage ± SEM of gross lesions of three or four pigs in each group at the indicated days. (**C**,**D**) Microscopic lung (**C**) and nasal turbinate (**D**) lesions of infected pigs are presented as the Mean ± SEM of lesions of three or four pigs in each group at the indicated days. The asterisks (*) represent a statistically significant difference between groups (*: *p* < 0.05; **: *p* < 0.01).

**Figure 2:**
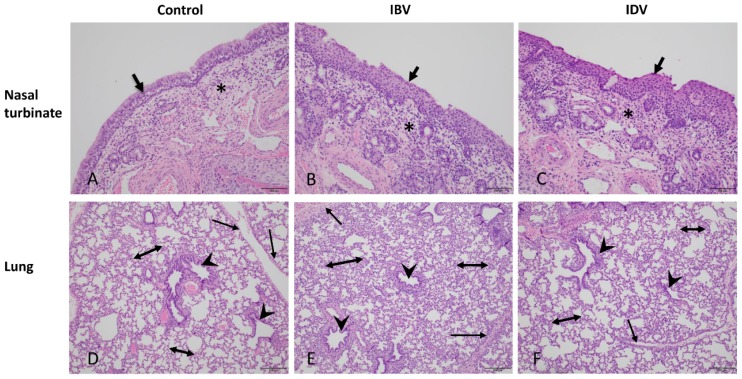
Histologic nasal turbinate and lung sections from pigs infected with either IBV or IDV at 8 days post-infection. *Top Row*: Nasal turbinate sections from control and infected pigs. Turbinate from control (**A**) have normal ciliated respiratory epithelium (arrow) and minimal inflammatory cells in lamina propria (asterisk). The IBV (**B**) or IDV (**C**) infected nasal turbinates have prominent segment of squamous epithelium/metaplasia (arrow) with infiltrating neutrophils through lamina propria and epithelium and increased lymphocytes in lamina propria (asterisks). *Bottom Row*: Lung sections from control (**D**) and infected pigs (**E,F**) taken after collection of lavage. Lungs infected with IBV (**E**) have prominent diffuse interstitial pneumonia with increase in cellularity of alveolar septae (double arrows), along interlobular septae (single arrows), and with prominent (though mild) peribronchiolar lymphocytes (arrowheads). Lungs infected with IDV (**F**) have milder diffuse interstitial pneumonia (increased thickening and cellularity of alveolar septae including along interlobular septae) and minimal peribronchiolar inflammation. Lungs of control pigs have minimal increased cellularity noted components. Bars for nasal turbinate and lung section are 100 and 200 µm, respectively.

Uninfected control pigs did not have any clinical signs through the length of the study with the exception of one pig that had a fever on day 6 post mock-infection. No obvious respiratory signs were observed in pigs infected with either the IBV or IDV. In contrast to the control pigs, 8 out of 10 pigs infected with IBV developed fever (temperature ≥ 104 °F) starting as early as 1 dpi and lasting from 1 to 6 days, while 9 out of 10 pigs infected with IDV displayed fever starting as early as 1 dpi and lasting from 1 to 5 days (Figure 1A). No macroscopic lung lesions were present in the control pigs, whereas macroscopic lung lesions were observed in pigs infected with either the IBV or IDV. The average lung lesion score was less than 2% in both IBV- and IDV-infected groups at 4, 6, and 8 dpi (Figure 1B). Nine out of ten pigs infected with IBV showed macroscopic lung lesions except one pig sacrificed at 8 dpi, while 8 out of 10 pigs infected with IDV exhibited gross lung lesions except one pig sacrificed at 4 dpi and one pig sacrificed at 6 dpi (Figure 1B). There was no significant difference in macroscopic lung lesion severity between IBV- or IDV-infected pigs. In addition, histologic lesions were evaluated from lung lobes and nasal turbinates collected from infected and uninfected control pigs at all necropsy dates. A very low lung lesion score was found in the control pigs in contrast to higher scores in both IBV- and IDV-infected pigs at each necropsy time point (Figure 1C). Slightly higher lung lesion scores were observed in IBV-infected pigs than those infected with IDV at all 3 (4, 6, and 8 dpi) tested time points, but a significant difference was only found at 8 dpi (Figure 1C). The lungs of all pigs infected with either IDV or IBV showed variable degrees of microscopic lung lesions, which ranged from minimal to mild peribronchiolitis and interstitial pneumonia/alveolitis (Figure 2E,F). Peribronchiolitis was characterized by increased numbers or layers of lymphocytes, macrophages, and rarely neutrophils around bronchioles and terminal airways and occasionally vessels. Patchy to diffuse areas of interstitial pneumonia/alveolitis were characterized by thickening and increased cellularity of alveolar septae around margins of the lobules and rarely degenerate and intact neutrophils, histiocytes, and/or sloughed pneumocytes in the alveolar lumen in both IBV- and IDV-infected pig lungs. No bronchiolar epithelial necrosis was observed in any of the pigs infected with either IBV or IDV (Figure 2E,F). Both IBV and IDV infections resulted in higher lesion scores in nasal turbinates of infected pigs than in controls although controls did have areas of mild inflammation. In contrast, a higher lesion score was found in the IDV-infected pigs at 4 and 6 dpi than in IBV-infected pigs (Figure 1D). Numerous regions of squamous epithelium/metaplasia associated with epithelial erosions and often neutrophilic infiltrates were present in turbinates from both IDV- and IBV-infected pigs (Figure 2B,C).

**Figure 3:**
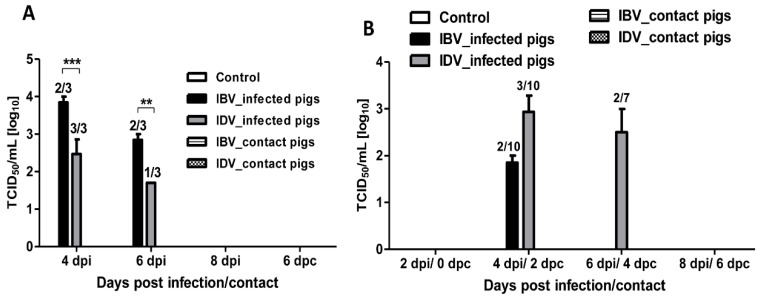
Virus titers in BALF and nasal swab samples collected from contact or infected pigs with IBV or IDV. (**A**) Mean of virus titers in BALF from contact or infected pigs with either IBV or IDV at indicated days. (**B**) Mean of virus titers in nasal swabs from contact or infected pigs infected with either IBV or IDV at indicated days. None of the contact pigs had virus in lung or nasal turbinates. Determination of virus titers was performed by calculating the 50% tissue culture infective dose (TCID_50_)/mL in Madin–Darby canine kidney (MDCK) cells using indirect immunofluorescence assay (IFA). The number of pigs with positive virus isolation out of the total number of tested pigs is presented above of each bar. The error bars represent SEM. The asterisks (*) represent a statistically significant difference between groups (**: *p* < 0.01, and ***: *p* < 0.001).

No virus was detected in BALF and nasal swab samples collected from the control pigs. Virus was detected in BALF samples collected from IBV- or IDV-infected pigs at 4 and 6 dpi; however, none of the IBV- or IDV-infected animals had detectable virus in the BALF at 8 dpi (Figure 3A). IBV was detected in BALF samples collected from two out of three infected pigs at both 4 dpi (10^4.0^ and 10^3.7^ TCID_50_/mL) and 6 dpi (10^3.0^ and 10^2.7^ TCID_50_/mL), while IDV was detected in BALF samples collected from all three IDV-infected pigs at 4 dpi (10^3.0^, 10^2.7^, and 10^1.7^ TCID_50_/mL) and from one out of three infected animals at 6 dpi (10^1.7^ TCID_50_/mL) (Figure 3A). A significantly higher virus titer was detected in BALF samples from IBV-infected pigs compared to those from IDV-infected pigs at both 4 dpi and 6 dpi (Figure 3A). Virus was detected in nasal swab samples collected from 2 out of 10 IBV-infected pigs at 4 dpi (10^2.0^ and 10^1.7^ TCID_50_/mL) only. In contrast, virus was detected in nasal swabs collected from 3 out of 10 IDV-infected pigs at 4 dpi (10^3.0^, 10^2.3^, and 10^3.5^ TCID_50_/mL) and from two out of seven infected pigs at 6 dpi (10^2.0^ and 10^3.0^ TCID_50_/mL) (Figure 3B). We also performed the specific real-time RT-PCR to detect IBV or IDV RNA in collected BALF and nasal swab samples of control, infected, and contact pigs. Results showed that all BALF samples collected from IBV- or IDV-infected pigs at 4 and 6 dpi were positive for the respective virus RNA except for one sample from an IBV-infected pig at 6 dpi. In addition, BALF samples from all four IDV-infected pigs collected at 8 dpi were positive by real-time RT-PCR, although no virus was isolated from these samples. A greater number of nasal swab samples was positive by the specific real-time RT-PCR as compared to BALF samples. IDV-positive swab samples were found at all time points (2, 4, 6, and 8 dpi) in IDV-infected pigs. In contrast, only three out of ten nasal swab samples collected from IBV-infected pigs were positive at 4 dpi (Table 1). These results indicate that both IBV and IDV can replicate and are pathogenic in pigs in the presence of IAV-specific antibodies.

### 3.2. Transmissibility of IBV and IDV in Pigs

To compare the transmissibility of IBV and IDV among pigs by direct contact, three sentinel pigs were commingled with IBV- or IDV-infected pigs at 2 dpi. None of the contact pigs in the IBV and IDV groups showed any respiratory clinical signs during the 6-day contact period. However, all contact pigs in the IBV- and IDV-infected groups showed fever, all lasting 1 or 2 days, with the exception of one contact pig in the IDV infection group that had a fever lasting 5 days (Figure 1A). Two out of three contact pigs in both IBV- and IDV-infected groups displayed some macroscopic lung lesions with an average lung lesion score at 6 dpc of 1.4% or 1.3% in the IBV- or IDV-infected groups, respectively (Figure 1B). No difference in lung lesion severity was observed between the two contact groups.

**Figure 4:**
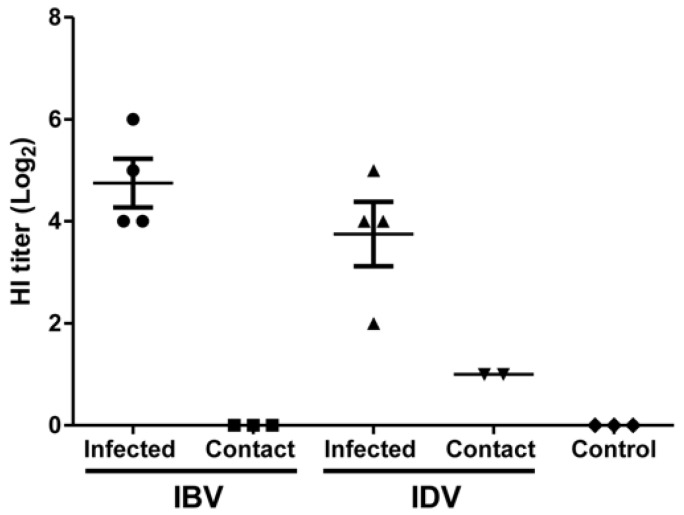
Hemagglutination inhibition (HI) titer of serum samples from contact or pigs infected with IBV or IDV at 8 dpi (6 dpc). HI assay was performed for serum samples collected from three control pigs, three contact pigs, and four infected pigs at 8 dpi based on a standard protocol. HI titer ≥ 20 is judged as positive.

No live virus was detected in BALF or nasal swab samples collected from contact pigs in both IBV- or IDV-infected group at all tested time points (Figure 3A,B). No IBV RNA was detected in BALF or nasal swab samples collected from contact pigs in the IBV-infected group at any tested time point (Table 1). However, IDV RNA was detected in nasal swab samples collected from contact pigs at 2, 4, and 6 dpc and in one BALF sample harvested from one contact pig at 6 dpc by specific real-time RT-PCR (Table 1). The other g two IDV-contact pigs harvested on 6 dpc were positive only in nasal swabs collected at 2 dpc, but not in BALF samples (Table 1). Seroconversion occurred in both IDV-infected and IDV-contact pigs as well as in IBV-infected pigs but not in IBV-contact pigs. No HI titer against both viruses was detected in control pigs. A higher HI titer was detected in the IBV infected pigs (Geometric Mean HI = 269) necropsied at 8 dpi when compared to the IDV-infected pigs necropsied on the same day (Geometric Mean HI = 135) (Figure 4). Interestingly, no contact pig in the IBV-infected group seroconverted, but two out of three contact pigs in the IDV-infected group seroconverted (HI titer of 20) (Figure 4). These results indicate that IDV is transmissible among pigs.

## 4. Discussion

Unlike other animal species susceptible to influenza virus infections, swine can be infected with all four genera of influenza viruses, IAV, IBV, ICV, and IDV. Pigs are known to play a critical role in IAV evolution as the mixing vessel for different IAVs to generate novel reassortant influenza strains with the potential to infect humans and cause pandemics [13,14]. The 2009 pandemic H1N1 virus is a good example of the critical role of pigs in the ecology of IAVs [12]. Although influenza IBV, ICV, and IDV viruses have been isolated in swine [25,26,27], the biology of these viruses in pigs is not well understood. In this study, we compared the pathogenicity and transmissibility of IBV and IDV after intratracheal and intranasal infection of pigs that previously had antibodies to both H3N2 and H1N1 IAVs. We clearly showed that IBV and IDV can replicate in pigs despite the presence of IAV-specific antibodies. Additionally, we confirmed that IBV- and IDV-infected pigs shed virus via the nasal cavities, consistent with previous findings [24,27]. Interestingly, IBV had a higher replication efficiency in the lower respiratory tract as compared to IDV, evidenced by a significantly higher virus titer in BALF samples in IBV-infected pigs. In contrast, IDV had a higher replication efficiency in the upper respiratory tract as more pigs shed virus for a longer period post-infection as compared to IBV. These results are in agreement with observed histopathological lesions in lung and nasal turbinate sections from pigs infected with either IBV or IDV. This finding most likely correlates with the distribution of virus receptors in pigs utilized by each virus. A previous study reported that IDV infection was limited to the upper respiratory tract, as evidenced by the lack of virus replication in lung samples from intranasally infected pigs [27]. We detected IDV in BALF of infected animals, indicating at least limited IDV replication in the lower respiratory tract. These different findings may be due to either the dissimilar inoculation routes (intranasal vs intranasal and intratracheal in the present study) or the different age of pigs used (11-weeks-old vs 5-weeks-old in the present study). Our results are in agreement with previous findings that IDV can be transmitted to contact pigs from infected animals [27]. In contrast, IBV was not transmissible among pigs in this study, as evidenced by a lack of IBV RNA, virus, and seroconversion in contact animals, although virus shedding was found in the infected pigs. This result differed from a previous study [24], which is most likely a result of the IAV-positive immune status of infected and contact animals in the present study. Nevertheless, we found that presence of IAV serum antibodies does not protect from IBV or IDV infection and replication in pigs.

Bovine respiratory disease complex (BRDC) is one of the most important multifactorial disease worldwide and costs the US cattle industry over one billion dollars annually [45]. IDV has recently been implicated as a contributor to BRDC and is commonly detected in cattle [45]. Similar to BRDC, PRDC has a serious economic impact on the global swine industry and is caused by simultaneous infection by multiple pathogens [46,47]. IAV has been shown to contribute to PRDC concurrently with other pathogens such as PCV2, PRRSV, and *Mycoplasma hyopneumoniae* [48,49,50]. In a previous study, we showed a higher prevalence of IBV antibodies in PRRSV-positive swine herds [24], suggesting a possible relationship between PRRSV and IBV infection. Although PRRSV is a main contributor to PRDC, it is possible that coinfection with IBV or IDV could exacerbate clinical signs of PRDC. Therefore, it is crucial to determine the pathogenesis of IBV and IDV in swine and their roles in multifactorial diseases such as PRDC.

The similarity between currently circulating influenza virus strains in human and swine populations emphasizes the importance of influenza research in swine. Pigs were shown to harbor IBV antibodies against lineages identical to the predominantly circulating IBV lineages in humans [24]. Phylogenetic analysis identified IBVs isolated from swine that may have originated in humans [25]. Taken together, these findings suggest the possibility of reverse zoonotic/ zoonotic transmissions of IBVs from humans to swine, and swine to humans, as has been shown for IAVs [14]. Since swine serve as a mixing vessel for different IAVs, similar reassortment events between different IBVs may also occur in pigs, which would allow to generate novel zoonotic IBVs. Although the likelihood of zoonotic transmission of IDV is not clear yet, several lines of evidence support the possibility of zoonotic IDV transmission. Firstly, a surveillance study of human serum samples found 1.3% of the tested group seropositive for IDV antibodies, with a much higher likelihood of IDV antibodies (95%) in individuals with occupational contact to cattle [6,27]. Furthermore, IDV has a broader cellular tropism than ICV, is known to infect humans, and has the ability to replicate in ferrets which are a well-known animal model for human influenza studies [27]. As both IBV and IDV can infect and transmit in pigs [24,27], the mixing vessel for IAVs, it is possible that novel IBV and IDV strains could be generated in pigs as well, potentially creating the risk for zoonotic transmission of novel strains to humans. Thus, more extensive studies are required to understand the pathogenicity and evolution of IBVs and IDVs in pigs to mitigate a possible threat to public health.

Taken together, we showed that both IBV and IDV can infect and replicate in pigs in the presence of IAV-specific antibodies. Despite the fact that both genera of influenza viruses do not cause severe respiratory disease in pigs, it is important to include these viruses in routine veterinary diagnostic swine panels as they could play a role in PRDC. In addition, more studies are needed to understand the molecular evolution of all genera of influenza viruses in pigs in order to protect animal and public health.

## Figures and Tables

**Table 1:** RT-PCR Ct values of the nasal swab and bronchoalveolar lavage fluid (BALF) samples from pigs infected with either IBV or IDV and from contact pigs.

dpi/dpc	Nasal Swab				BALF			
	IBV		IDV		IBV		IDV	
	Infected	Contact	Infected	Contact	Infected	Contact	Infected	Contact
**2 dpi**	ND (0/10)	ND (0/3)	34.88 (5/10)	ND (0/3)	NA	NA	NA	NA
**4 dpi (2 dpc)**	34.36 (3/10)	ND (0/3)	30.36 (8/10)	35.89 (3/3)	28.84 (3/3)	NA	24.10 (3/3)	NA
**6 dpi (4 dpc)**	ND (0/7)	ND (0/3)	31.30 (7/7)	35.25 (1/3)	29.08 (2/3)	NA	28.33 (3/3)	NA
**8 dpi (6 dpc)**	ND (0/4)	ND (0/3)	35.18 (2/4)	30.02 (1/3)	ND (0/4)	ND (0/3)	31.54 (4/4)	34.44 (1/3)

Results are presented as average of Ct values of the nasal swab and BALF samples from infected or contact pigs at indicated days. Sample analysis was performed in duplicates. Numbers in parentheses indicate the number of pigs positive by real-time RT-PCR out of total numbers of pigs. ND, not detected. NA, not applicable.
